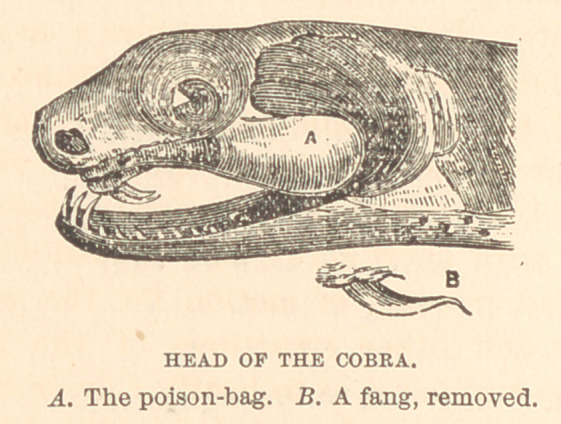# Seventh Annual Session of the Maryland State Dental Association

**Published:** 1890-05

**Authors:** 


### SEVENTH ANNUAL SESSION OF THE MARYLAND STATE DENTAL ASSOCIATION.

(Continued from page 229.)

The annual session was held on December 5 and 6, 1889, at the St. James Hotel, Baltimore, the president, E. P. Keech, M.D., D.D.S., in the chair.

*Friday, December 6, 1889.—Afternoon Session.*

Report from Committee on Anatomy, Physiology, and Histology, consisting of Drs. R. B. Winder, *chairman*, Richard Grady, and W. H. Montell.

*Dr. R. B. Winder.*—As chairman of the committee of the Section on Physiology, Surgery, Histology, and Microscopy, I will have to report that, by some means or other, the impression was made on our minds that anatomy was included and placed at the head of the list of subjects for our consideration. So it was thought wise to commence with this fundamental branch of all medical studies, and offer a paper, this year, on anatomy as a starting-point, to be followed next year by one on physiology; the year after, by one on surgery; and later still, by one on histology and microscopy. We beg leave to apologize for our misconceptions, but, in all due deference, would suggest that anatomy properly belongs to this section, and should be added thereto. At the committee meeting a general plan of dealing with this subject was agreed upon, and Dr. Richard Grady generously consented to work up the matter, and present what your committee have to report, and to him belongs the credit of preparing this essay, which to him, no doubt, has been a very laborious pleasure. We sincerly hope that his efforts will be duly appreciated.

Richard Grady, M.D., D.D.S., then read the following paper on Anatomy.

The Committee on Anatomy, Physiology, and Histology, as now constituted, in order to fill an important gap which has existed since the Association was founded, has confided to me the difficult task of preparing the report for this year, it being understood that the other members will, if reappointed, severally speak on Physiology and Histology at future meetings. A twenty-minute paper on anatomy must of necessity be rigidly eclectic, when many volumes are given to its full exposition. I have intentionally abstained from burdening the text with references, and have freely used the works at my command; in some cases paraphrasing, or even adopting *verbatim*, the author's language, when it suited my purpose.

The dental organs, through the digestive function, hold an important relation to the whole body. Dentistry, therefore, includes the sciences which lie at the foundation of all medical art. The anatomy, physiology, and pathology of dentistry should, therefore, differ in no respect from that of medicine; in fact, human anatomy is a distinct branch of study in connection with physiology, pathology, surgery, and therapeutics; and a dentist's knowledge of these fundamental sciences admits of no limitation; in most cases his knowledge is insufficient to give full value to the subsequent lessons of experience.

During the primitive ages of the world anatomy, which is now one of the most important branches of natural science, was little cultivated as a science, and hence the art of surgery was undeveloped. The ancients, ignorant alike of the anatomy and diseases of the human body, supposed that manual dexterity was all that the surgeon required; and the well-known etymology of the word *surgery*—"hand-work"—conveys that idea. In our own day, in our own city, a like sentiment has been expressed, it being claimed that dentistry is ninety-nine and a half per cent. mechanical. The surgeon, during the last half of the century, has not been considered inferior to the physician. He must be his equal in medicine to become eminent; and besides possessing a knowledge of anatomy, the M.D., for his major operations, and the D.D.S., for his minor operations, should have, to use the language of Celsus, "a hand steady, expert, and never tremulous, and an intrepid mind." To guide them in their delicate and difficult operations, a proper knowledge of the relations of organs to each other, such as the positions, forms, dimensions, structure, and peculiarities of nerves, vessels, muscles, glands, and membranes is necessary. They must know *where to cut* and *what to avoid* in operating on the living body, for the life of the patient might be jeopardized if they were not well acquainted with the anatomy of the vital organs.

It is difficult to determine the date at which the science of anatomy began to be cultivated. It is probable, says Galen, that Æsculapius, who excelled in the treatment of wounds, dissected animals for the instruction of his pupils. Although among the Jews the touching of a dead body involved ceremonial uncleanness, they did not entirely neglect anatomy.[1] They counted two hundred and forty-eight bones and three hundred and sixty-five veins and ligaments, which division, it is said, has relation to the two hundred and forty-eight precepts of the Mosaic law that *command* and the three hundred and sixty-five that *forbid.* Hippocrates was the first author who treated anatomy as a science. He caused a skeleton of brass to be cast, which he consecrated to the Delphian Apollo, with a view of transmitting to posterity proofs of the progress he had made and stimulating others to the study of anatomy. Aristotle possessed nothing certain on the subject beyond what could be drawn from the probable resemblance of the corresponding parts of other animals. He first gave the name *aorta* to the great artery. Human bodies were first dissected B.C. 300, and it is said that some condemned to death were dissected while they were still alive. Galen (A.D. 131) dissected apes, as being most like human subjects, though he occasionally obtained bodies of children exposed in the fields, or of persons found murdered, which, how-

---

[1] 1. That the Jews paid attention to anatomy is shown by the fact that Hebrew possesses names for all the organs and their parts. Cf. "Das Arabische und Hebräische in der Anatomie," Von Dr. Joseph Hyrtl, Emeritus Professor of Anatomy at the University of Vienna (Vienna, 1879). 2. There is recorded in the Talmud a case of dissection. In "Bechoroth," 45, a, it is related that a certain disreputable woman was condemned to be burned, but that her body was given to the pupils of Rabbi Ishmael, who discovered that a woman's body contains two hundred and fifty-two, and not two hundred and forty-eight, bones. 3. The ancient Jews had simple and effective remedies for diseases, which contrasted strongly with the almost universal use of magical formulæ employed by other nations. Cf. Dr. Joseph Bergel, "Die Medizin der Talmudisten nebst einer Anhange die Anthropologie der alter Hebraer" (Leipsic, 1885). 4. The sanitary regulations concerning the food drew the attention of the Hebrews to anatomy. Thus a cow must not only be slaughtered according to certain regulations, but its lungs must afterwards be examined, and if any trace of pleuro-pneumonia be found, the flesh of the animal was considered unfit to be eaten.

ever, he was obliged to dissect in secret. There was at this time no regularly prepared skeleton, as there was a Roman law forbidding the use of dead bodies. Galen also collected the works of his predecessors. He first showed that arteries in the living animal contain blood, not air alone; but it did not occur to him to notice the circulatory movement of the blood; that was reserved for Harvey fifteen centuries later. As you know, the ancients supposed the arteries to contain "spirits" or air, because, when cut open in the dead body, these vessels do not collapse, as a vein would, but stand open, allowing the air to pass in. It was this circumstance which led the old anatomists to believe that the arteries also contained air during life.

Anatomy made small progress among the Arabs, which is accounted for by the Mohammedan religion prohibiting contact with dead bodies; but the Arabians cultivated the natural sciences in the Middle Ages when they were neglected by the Christians. When a great Arabian physician (Rhazes, 852) was about to be operated on for cataract, he discovered that the surgeon was ignorant of the structure of the eye, and refused to submit to the operation.

Anatomy was now neglected for a long period, till the King of Sicily, in the thirteenth century made a law forbidding any one to practise surgery without having first acquired some knowledge of anatomy. He founded a chair, at the solicitation of his chief physician, where the science was demonstrated for five years. Students from all parts crowded to it, and some time after a similar school was established at Bologna, where one of the surgeons (Vigo) boasted of having dissected more than one hundred subjects. Reports were circulated that he had dissected living Spaniards, and he fled or was exiled. Another surgeon (Vesalius, 1514) had the misfortune to open the body of a young Spanish nobleman whose heart was found beating, and he was obliged to make a pilgrimage to Jerusalem. The first work in English on anatomy was published in 1548.

In the seventeenth century progress was rapid. Harvey, in 1619, discovered the circulation of the blood, and the microscope was employed to detect the structure of minute vessels. In 1622 the existence of lymph-vessels was discovered and demonstrated. The glandular vessels were investigated by Wharton, while Malpighii and (in the following century) the illustrious Ruysch, by the use of injections and the aid of the microscope, gave a new impulse to the research in minute structures. Eminent names in the history of anatomy are numerous in the eighteenth century. We find them in Italy, which still retained its former pre-eminence; in France, including Bichat, the founder of general anatomy; in Germany, Haller and Meckel prepared the way for greater achievements in the nineteenth century; in Great Britain, Hunter and Charles Bell contributed to the progress of the science. On the boundaries of the two centuries we find names nearly all connected with practical medicine, which was benefited by the studies in anatomy. Many of the great discoveries of comparative anatomy and general anatomy have been made in the present age; and the systematic study and development of minute anatomy dates from the improved construction of the compound microscope. The necessity of a union of theory and practice has led to the zealous study of pathological anatomy by modern scholars. Eminent contributors to comparative anatomy are familiar to you, as are also the names of those who have studied it with especial reference to physiology. First Italy, then Holland, Denmark, Sweden, Germany, France, England, and America have furnished them, but popular prejudices have hindered free dissection of human bodies in medical schools until a very recent date.

The word "anatomy" is still commonly used to signify "human anatomy." Almost all begin the study of the science as medical and dental students with the dissection of the human body, *and most end there;* but no special anatomy can be rightly and fully understood save on the basis of general science, of which it is an integral part. The reason lies in the diversities of organic structure being subordinated to a principle of unity.

Without some knowledge of comparative anatomy it is impossible to understand the beautifully progressive development of organization. It is necessary even for the full comprehension of the uses of many parts of the human body, which, apparently rudimentary and useless in man, are highly developed in other animals. This science is also the basis of physiology and the natural classification of animals. On a subject so vast as this, comprehending the whole range of animal life, it will be impossible here to give anything but the briefest sketch, making it referable to the organs of alimentation and digestion, and especially to the teeth.

In man the upper jaw-bones contain all the upper teeth; but in the lower animals the incisors are contained in the intermaxillary, a persistence of separation which may be detected in the human fœtus. No animal but man has a chin. In all below him the interior arch of the lower jaw is convex vertically and retreating at its lower margin.

The range of the subject of dental anatomy turns upon the meaning which is attached to the word "tooth." Most vertebrates and a great many invertebrates have certain hard masses in or near to the orifice of the alimentary canal,—*i.e.*, the mouth. By these hard masses, sometimes of bony and sometimes of horny nature, various offices in connection with the prehension and comminution of food are performed, and to them the term "teeth" is applied. In many animals teeth have come to be used for other purposes, such as for sexual warfare; but it can hardly be doubted that teeth have primarily to do with the nourishment of their possessor. No one can doubt, whether from the comparison of adult forms, or from a study of the development of the parts, that the teeth of the shark correspond to the teeth of other fish, and these again to those of reptiles and mammals. It may be clearly demonstrated that the teeth of the shark are nothing more than highly-developed spines of the skin, and therefore it is inferred that all teeth bear a similar relation to the skin. This is what is meant when teeth are called "dermal appendages," and are said to be perfectly distinct from the internal bony skeleton of the animal. The teeth of the shark, and of many other creatures, remain embedded in tough, mucous membrane, and never acquire any connection with the bone. Indeed, all teeth are developed from a part of the mucous membrane, and any connection which they may ultimately get with the bone is a secondary matter. It has been well expressed by Dr. Harrison Allen, in his "Anatomy of the Facial Region," that "if the hairs of the scalp were to be inserted into the skull, or of the moustache into the upper jaw, we should express great astonishment, yet such an extreme proposition is no more remarkable than what is seen to take place in the jaws." Again, "the feathers of certain birds making impressions on the radius, the whalebone pendent from the roof of the mouth, are examples of this same association of tegumentary appendages with the bones." In their simpler forms, then, the teeth are met with as very numerous spines. In many fish the teeth, though more specialized, are scattered over almost every one of the numerous bones which form part of the walls of the mouth and pharynx; in reptiles they are much more limited in position, and in mammals are absolutely confined to the intermaxillary, superior maxillary, and lower maxillary bones. In fish and reptiles it is the exception for the teeth in different parts of the mouth to differ markedly from each other; in mammals it is the rule.

There is no organ so characteristic of the animal, as distinguished from the vegetable, as an internal digestive cavity for the conversion of organic substances into nutritive material. In the sac-like polyps (*e.g.*, the animals that form coral) the food is introduced into the simple stomach and dissolved without any mechanical division; in the higher invertebrates, and all the vertebrates, there is a distinct mouth, an apparatus for mastication, a stomach for digestion, and an intestine from which the nutrient matters are absorbed and the useless materials are expelled. In vertebrates the teeth are confined to the cavity of the mouth, and generally to the jaws, none being found in the stomach. In serpents, which feed on living prey, the sharp, conical teeth are directed backward and the bones to which they are attached are freely movable, enabling them to swallow animals considerably larger than themselves; the venomous class have in the front of the upper jaw two (Professor Winder has seen more) long, curved fangs, communicating by a canal or a groove with the poison-gland behind and below the orbit; the muscles which close the jaws press the venom into the wound made by the teeth; in the rattlesnake these fangs are movable, and may be bent backward in a fold of the gum when not in use; behind the ones actually employed, there are rudiments of others which soon complete the terrible armature, if one fang happens to get broken.[1]

HEAD OF THE COBRA.

*A.* The poison-bag. *B.* A fang, removed.

The bill furnishes to the zoölogist as good characters for the classification of birds as do the teeth for that of mammals; its exterior and the sharp edges are covered with solid horn, but it never has any true teeth, so that there is no proper mastication in this class.

---

[1] The cut affords a view of the poison-gland, and the backward position of the fangs. The instant the wound is inflicted, the roots of the fangs press behind on the venom-bag, causing the fluid to run down a groove or channel in each fang, by which means the *virus* is carried directly into the punctures made.

The existing kinds of vertebrates constitute part only, perhaps but a small proportion, of those which have lived. More than one-half the groups of the class indicated by osteal and dental characters have perished; and it is only by petrified fæces or casts of the intestinal canal, by casts of the brain-case, or by correlative deductions from characters of the petrified remains, that we are enabled to gain any glimpses of the anatomical conditions of the soft parts of such extinct species: by such light some of the perishable structures of these animals are indicated in works of comparative anatomy.

As vertebrates rise in the scale, and the adaptive principle predominates, the law of correlation, as enunciated by Cuvier, becomes more operative. In the jaws of the lion, for example, there are large canines, formed to pierce, lacerate, and retain its prey. There are also compressed, trenchant, flesh-cutting teeth which play upon each other like scissor-blades in the movement of the lower upon the upper jaw. The lower jaw is short and strong; it articulates with the skull by a condyle received into a corresponding concavity, forming a close-fitting joint which gives a firm attachment to the jaw, but almost restricts it to the movements of opening and closing the mouth.

The jaw of the carnivora develops a plate of bone of breadth and height adequate for the implantation of muscles with power to inflict a deadly bite. These muscles require a large extent of surface for their origin from the cranium with concomitant strength and curvature of the zygomatic arch, and are associated with a strong, occipital crest and lofty dorsal spines for vigorous uplifting and retraction of the head when the prey has been gripped. The limbs are armed with short claws and endued with the requisite power, extent, and freedom of motion for the wielding of these weapons. These and other structures of the highly-organized carnivora are so co-ordinated as to justify Cuvier in asserting that the "form of a tooth gives that of the condyle, of the bladebone, and of the claws, just as the equation of a curve evolves all its properties; and exactly as, in taking each property by itself as the base of a particular equation, one discovers both the ordinary equation and all its properties, so the claw, the bladebone, the condyle, and all the other bones individually give the teeth or are given, thereby reciprocally; and in commencing by any of these, whoever possesses rationally the laws of the organic economy will be able to reconstruct the entire animal. The law of correlation receives as striking illustrations from the structures of the herbivorous mammal. A limb may terminate in a thick, horny hoof; such a foot serves chiefly, almost exclusively, for locomotion. It may paw the ground, it may rub a part of the animal's hide, it may strike or kick; but it cannot grasp, seize, or tear another animal. The terminal ungulate phalanx gives, so Cuvier declares, the modifications of all the bones that relate to the absence of a rotation of the foreleg, and those of the jaw and skull that relate to the mastication offered by broad-crowned complex molars.

DISCUSSION OF REPORT OF COMMITTEE ON ANATOMY, PHYSIOLOGY, AND HISTOLOGY, PRESENTED BY DR. GRADY.

*Dr. B. Holly Smith.*—I think that Dr. Grady deserves much credit for the able, clear, and concise paper which has just been read. It is customary in our schools to impart a knowledge of special anatomy or of those branches of it that may be of special interest to the dentist; but being of an intricate nature, the study is one that requires a student to devote his whole time to it if he would become proficient in it. This it is not always possible for him to do, and the consequence is that, as a rule, the members of our profession, while possessing a general knowledge of anatomy, do not make any great pretensions as surgical or anatomical experts. Therefore, when an operation is to be performed requiring the location of an important artery or nerve, an appointment is made and sufficient opportunity is afforded meanwhile for the operator to brush up his knowledge and prepare himself for the special operation.

Unless afforded opportunity to mature our thoughts upon the subject, I do not think we are capable of adding anything to the anatomical history which has been detailed, and which could have been prepared only after the most laborious and patient investigation. I can only say that the study of anatomy is an absorbing and fascinating one. Certainly mathematics cannot be more attractive to its devotee than is anatomy to one who persistently and patiently devotes himself to acquiring a knowledge of it. I have only to say, as an apology for my inability to lead the discussion in a direction so interesting as the one here indicated, that we as specialists, while having a knowledge of anatomy, do not possess any special qualification for an intelligent discussion of the subject.

*Dr. R. B. Winder.*—Anatomy, as is well known, is like mathematics or the multiplication table in that it is a subject that does not admit of much difference of opinion or controversy. The paper just presented received my endorsement as the chairman of the committee on the subject, and I have nothing to add to it. It is an accumulation of facts that have been compiled by anatomists, and I believe its statements to be absolutely correct. There are many interesting points connected with anatomy which are not embraced within the scope of the paper and which would not be fit subjects for discussion here.

*Dr. A. J. Volck.*—A paper like the one just presented is understood to be literally accurate in its statements. I therefore beg pardon of my friend (Dr. Grady) to allow me to correct what I consider to be one error in it. I refer to the statement in regard to the rattlesnake. The paper speaks of the rattlesnake as shutting its mouth when it bites. The doctor is mistaken in regard to that. The rattlesnake does not shut its mouth when it strikes; and what is true of the rattlesnake, in this respect, is equally true as to other poisonous snakes. The teeth lie flat in the mouth during quiescence, and are only erect when the snake strikes. This is a peculiarity not of the rattlesnake alone but of all venomous snakes.

*Dr. R. Grady.*—If the statement of the paper is not accurate, I am grateful for the correction.

*Dr. R. G. Winder.*—I do not think that Dr. Volck is correct in assuming that the language of the paper is as he has stated it. That the rattlesnake strikes and does not bite, so far as the use of its poisonous fangs are concerned, is well known. If the paper contains a statement to the contrary, the error escaped not only my notice, but that of the other members of the committee.

*Dr. R. Grady.*—The statement was based not upon any personal knowledge of my own, but simply upon what I regarded as a matter of record. I have not here the authorities with which to support the statement, but I have preserved the original extracts which I made and will refer to them later. Upon consulting one of our members who, I thought, was familiar with the peculiarities of the rattlesnake, he was unable to give me sufficient light on the point, and I was obliged to rely upon the books. I will either verify the statement or, if it is not correct, expunge it.

*Dr. A. J. Volck.*—That part of the report to which I refer states that "the venomous class of serpents have in the front of the upper jaw two or more long, curved fangs, communicating by a canal or a groove with the poison-gland behind and below the orbit; the muscles which close the jaws press the venom into the wound made by the teeth; in the rattlesnake these fangs are movable, and may be bent backward in a fold of the gum when not in use."

That is what I say. They are bent backward in all poisonous snakes. In the rattlesnake these fangs are movable.

My object is to show that it made an exception in favor of the rattlesnake as the only snake whose teeth are movable and lie back in the mouth during quiescence; my own contention being that this peculiarity is applied alike to all poisonous snakes.

*Dr. Edward Nelson.*—I endorse the general scope of the paper, inasmuch as, instead of being confined to the extraction of teeth and the making of artificial teeth, it has taken a wider range upon the theory that dentists should be educated in all the branches pertaining to their specialty. Regarding it in that light, I think the paper is ably written and is worthy of the endorsement of the Association.

*Dr. W. A. Mills.*—I must take exception to the statement of the paper in reference to the embalming of the dead by the Jews. I believe that the Jews are strictly opposed to embalming, though there was an exception in their case, as we learn from biblical sources, and that was when Jacob was embalmed and carried from Egypt.

*Dr. Volck.*—He was embalmed by the Egyptians.

(To be continued.)

## Figures and Tables

**Figure f1:**